# Short- and Long-Term Mortality Rates of Elderly Acute Kidney Injury Patients Who Underwent Continuous Renal Replacement Therapy

**DOI:** 10.1371/journal.pone.0167067

**Published:** 2016-11-22

**Authors:** Harin Rhee, Keum Sook Jang, Jong Man Park, Jin Suk Kang, Na Kyoung Hwang, Il Young Kim, Sang Heon Song, Eun Young Seong, Dong Won Lee, Soo Bong Lee, Ihm Soo Kwak

**Affiliations:** 1 Department of Internal Medicine, Pusan National University School of Medicine, Busan, Republic of Korea; 2 Biomedical Research Institute, Pusan National University Hospital, Busan, Republic of Korea; 3 Department of Nursing, Pusan National University Hospital, Busan, Republic of Korea; University of Nottingham, UNITED KINGDOM

## Abstract

**Background:**

The world’s population is aging faster and the incidence of acute kidney injury (AKI) needing continuous renal replacement therapy (CRRT) is increasing in elderly population. The outcome of AKI needing CRRT in elderly patients is known to be poor. However, the definitions of elderly used in the previous literatures were diverse and, there were few data that compared the long-term mortality rates of these patients with middle aged patients. This study was aimed to evaluate this issue.

**Methods:**

This study was a single-center, retrospective cohort study of patients who underwent CRRT from January 2013 to December 2015. The patients were divided into the following four age cohorts: middle-aged (55–64), young-old (65–74), middle-old (75–84), and old-old (≥85). The short- and long-term mortality rates for each age cohort were compared.

**Results:**

A total of 562 patients met the inclusion criteria. The short-term mortality rate was 57.3% in the entire cohort. Compared with the middle-aged cohort, the middle-old cohort (HR 1.48 (1.09–2.02), p = 0.012) and the old-old cohort (HR 2.33 (1.30–4.19), p = 0.005) showed an increased short-term mortality rate along with an increased SOFA score, acidemia and a prolonged prothrombin time. When we analyzed the long-term mortality rate of the 238 survived patients, the middle-old cohort (HR 3.76 (1.84–7.68), p<0.001), the old-old cohort (HR 4.40(1.20–16.10), p = 0.025), a lower BMI, the presence of liver cirrhosis, the presence of congestive heart failure and a history of sepsis were independent risk factors for the prediction of long-term mortality.

**Conclusion:**

Compared with the middle-aged cohort, the middle-old and the old-old cohort showed an increased short-term and long-term mortality rate. However, in the young-old cohort, neither the short-term nor the long-term mortality rate was increased.

## Introduction

The world’s population is aging faster than ever, and life expectancy is increasing quickly. According to the WHO reports, global life expectancy at birth for both sexes was 65.3 years old in 1990 and has increased by 6.2 years to 71.5 years old in 2013 [1]. In developed countries, life expectancy exceeded almost 80 years old in 2013 [1]. Traditionally, old age was defined as the chronological age of 65 years or more. However, with increasing life expectancy, the traditional definition for an elderly population has become too heterogeneous, and a more detailed classification is needed. Recently, elderly patients were sub-divided into three life-stage groups: the young-old (aged 65–74), the middle-old (aged 75–84), and the old-old (aged over age 85) [2].

Acute kidney injury (AKI) is common in geriatric patients with acute illness [3, 4], and the incidence of AKI in elderly patients is increasing [5, 6]. Elderly patients are vulnerable to AKI because they have multiple comorbidities, polypharmacy and age-related structural, functional and hemodynamic changes in their kidneys [5, 7]. Furthermore, because of these conditions, elderly AKI patients are at a higher risk of hemodynamic instability and are therefore more likely to undergo continuous renal replacement therapy (CRRT) [4, 8]. Nevertheless, the decision to initiate CRRT in elderly persons is difficult and complex because these patients may not fare well on this aggressive, expensive and life-sustaining type of therapy [9–11]. To date, CRRT outcome data on elderly patients are scarce [4, 7, 12], and detailed information regarding CRRT outcomes in elderly subgroups has not been reported. Thus, it is crucial to assess the therapeutic efficacy of CRRT in the elderly population according to age subgroups.

In this study, we aimed to compare the short- and long-term mortality rates among different age subgroups and to evaluate the predictive factors for short- and long-term mortalities of the elderly population.

## Methods

### Patients

This investigation was a single-center, retrospective cohort study based on consecutively collected data from AKI patients who underwent CRRT in the intensive care unit (ICU) from January 2013 to December 2015. We included all adult patients aged more than 54 years old with an emergency ICU admission or elective post-operative ICU admission who had an expected ICU stay of more than 24 hours. For the purposes of analysis, the patients were divided into four age cohorts: the middle-aged (aged 55–64), the young-old (aged 65–74), the middle-old (aged 75–84), and the old-old (aged over 85) cohorts.

We received approval to perform anonymous analyses of routinely collected clinical data with a waiver of informed consent from the Pusan National University IRB Committee [E-2016014]. Due to the retrospective study design, the informed consent was exempt from review according to the IRB. Each patient record was anonymized and de-identified prior to analysis.

### Data Collection

We collected baseline demographic data, the comorbidities of each patient and biochemical data at the time of CRRT initiation. All the comorbidity data were collected based on the medical chart review and the presence of each disease was defined based on the description in the medical record. To assess the degree of organ failure and disease severity at the time of CRRT initiation, we reviewed data regarding PaO2/FiO2, serum creatinine, serum bilirubin, serum platelet count, mean arterial pressure, the use of vasopressor and the Glasgow coma scale (GCS) score; then, we calculated the sequential organ failure assessment (SOFA) score [13, 14]. The amount of urine output was calculated as the sum of urine output for 6 hours prior to the CRRT initiation divided by the body weight and hour. We reviewed each patient’s CRRT-associated treatment history, such as the CRRT initiation time, actual delivered dose and total duration of CRRT operation. The CRRT initiation time was assessed as the time from the recognition of AKI to CRRT application. The actual delivered dose was calculated using the effluent flow rate and corrected for the percentage of predilution.

### Patient Outcome

We defined short-term patient mortality as a death during the hospital stay or death within one week after hospital discharge. Long-term mortality was defined as a death at least one week after hospital discharge. Patient mortality was identified with a medical chart review or phone call.

### Statistical Analysis

The data were analyzed using SPSS for Windows, version 17.0 (SPSS Inc., Chicago, IL, USA) and R, version 3.2.2 (R Foundation for Statistical Computing, Vienna, Austria). For continuous variables, the mean ± standard deviations were used to describe normally distributed data, and other data were described using the median. Differences among the four age cohorts were tested using a one-way ANOVA test for continuous variables and a chi-square test for categorical variables. The analyses of the short- and long-term mortality rates for different age cohorts were performed by means of the log-rank test, and the results are presented as a Kaplan-Meier cumulative incidence plot. To identify the predictive factors for short- and long-term mortalities, we used Cox regression analyses. The variables included in the equations were chosen based on the results of the univariable analyses, such that each parameter demonstrated an association with short- or long-term mortality (p<0.1). We also included variables based on empirical evidence from previous studies that demonstrated a definitive association of short- and long-term mortality with an independent variable. Factors that are used in the SOFA score [13] were excluded during the adjustment to avoid overlapping. The following factors were adjusted in the multivariable Cox regression analysis for the short-term mortality rate: age cohort, sex, the presence of DM, HT, chronic kidney disease, congestive heart failure, cancer, immuocompromised host, the SOFA score, total protein, serum albumin, pH and prothrombin time; for the long-term mortality rate, the factors were as follows: age cohort, sex, BMI, the presence of congestive heart failure, liver cirrhosis, chronic kidney disease, prothrombin time and a history of sepsis. We calculated optimal cut off points for age that predicted short- and long-term mortality using a time to event approach by employing maximally selected log-rank statistics with the maxstat package in R 3.2.2. P values less than 0.05 were considered to be statistically significant.

## Results

### Baseline Characteristics in the Different Age Cohorts

A total of 562 patients were included in the study (S1 Fig). The mean age was 70.43±8.22 years old, and 63.2% of the patients were male. The most common cause of CRRT initiation in the elderly patients was AKI with septic shock (46.4%) and the second most common cause was AKI with medically uncontrolled pulmonary edema (37.7%). The other causes of CRRT initiation were summarized in Table 1. When we divided the patients according to the age cohort, 151 patients were classified as middle-aged, 229 patients as young-old, 162 patients as middle- old and 20 patients as old-old. The percentage of males decreased with increasing age. Height and weight showed decreasing trends with increasing patient age; however, the mean BMI was not different among the different age cohorts. A total of 96.4% of the patients had one or more comorbidities and hypertension was the most common comorbid disease in this cohort. The prevalence of hypertension, old myocardial infarction, atrial fibrillation and chronic kidney disease showed increasing trend with aging. The prevalence of liver cirrhosis decreased with aging. CRRT was performed as continuous veno-venous hemodiafiltration (CVVHDF) mode, followed by the protocol of our clinic as described in our previous research [15]. The CRRT initiation time and actual delivered dose were not different among the different age cohorts. The detailed results of the baseline characteristics are summarized in the Table 2.

**Table 1:** Main causes of CRRT initiation.

Causes	%
AKI with septic shock	46.4 (244/562)
AKI with acute brain injury	5.7 (32/562)
AKI without septic shock or acute brain injury	
Acidemia	3.9 (22/562)
Acute pulmonary edema	37.7 (212/562)
Hyperkalemia	1.8 (10/562)
Uremic complications	1.2 (7/562)
Etc	
Drug intoxication	1.4 (8/562)
Rhabdomyolysis	3.4 (19/562)
Tumorlysis syndrome	1.4 (8/562)

Abbreviations: AKI, acute kidney injury

**Table 2:** Patient characteristics, biologic and treatment data according to age groups.

	Total (N = 562)	55≤Age<65 (N = 151)	65≤Age<75 (N = 229)	75≤Age<85 (N = 162)	85≥ (N = 20)	P
**Dermographics**						
Age, year	70.43±8.22	59.79±3.01	70.11±2.81	78.81±2.74	86.55±1.76	<0.001
Male, %	63.2	72.2	64.6	54.3	50.0	0.006
BMI.kg/m^2^	23.00±3.98	23.06±4.87	23.29±3.80	22.66±3.30	22.14±3.43	0.350
**Comorbidities**						
Diabetes, %	44.3	44.4	44.5	46.3	25.0	0.350
Hypertension,%	73.1	64.9	73.4	82.1	60.0	0.003
CHF,%	40.4	41.1	39.3	41.4	40.0	0.977
Old CVA,%	24.4	21.2	24.9	27.2	20.0	0.625
Old MI,%	25.8	14.6	28.4	32.7	25.0	0.002
Atrial fibrillation,%	21.9	13.2	22.7	28.4	25.0	0.013
CKD,%	32.4	20.5	34.5	41.4	25.0	0.001
LC,%	8.2	15.2	8.7	1.9	0.0	<0.001
Malignancy,%	22.4	21.2	24.9	19.8	25.0	0.643
Immunocompromised, %	7.5	10.6	5.2	7.4	10.0	0.265
**Disease severities**						
MAP, mmHg	79.51±14.92	82.60±15.25	79.23±14.80	77.27±14.42	77.61±14.80	0.014
Ventilator, %	55.7	58.3	56.3	53.1	50.0	0.764
Vasopressor, %	61.4	57.0	64.2	59.3	80.0	0.155
SOFA score	10.18±3.63	10.44±3.60	9.94±3.87	10.20±3.38	10.75±3.14	0.520
Urine output, cc/kg/hr	0.67±0.92	0.75±1.12	0.67±0.88	0.64±0.82	0.37±0.34	0.791
Body Temperature,°C	37.04±0.83	37.07±0.89	37.11±0.81	36.95±0.80	36.73±0.62	0.094
Septic AKI, %	43.4	38.4	41.9	512	35.0	0.110
**Laboratory value**						
WBC,/uL	14.85±11.00	13.97±11.22	15.47±12.11	15.15±9.49	11.95±6.20	0.361
Hb, g/dL	10.65±5.35	10.59±2.69	10.76±5.69	10.64±6.81	9.83±2.18	0.897
Hct,%	30.66±7.48	31.50±10.01	30.70±6.53	29.97±5.88	29.25±6.53	0.258
PLT, 10E3/uL	157.98±129.23	151.27±122.32	166.39±160.74	155.98±83.49	128.92±31.10	0.489
TP, g/dL	5.55±1.01	5.58±1.10	5.59±1.01	5.46±0.91	5.43±0.93	0.559
Albumin,g/dL	2.96±0.63	3.02±0.70	2.96±0.61	2.91±0.58	2.98±0.69	0.451
pH, mmHg	7.31±0.12	7.29±0.13	7.31±0.12	7.31±0.12	7.32±0.12	0.195
BUN,mg/dL	56.24±31.19	56.32±31.84	54.39±29.18	57.76±33.13	64.52±32.83	0.459
Creatinine,mg/dL	3.33±2.45	3.55±2.67	3.20±2.09	3.23±2.45	3.89±4.01	0.376
Na,mmol/L	137.39±7.68	135.79±8.04	138.21±7.61	137.65±7.20	138.13±8.29	0.022
K, mmol/L	4.47±1.00	4.54±1.09	4.34±0.90	4.53±0.99	5.01±1.29	0.011
PT, INR	1.66±0.89	1.66±0.73	1.74±1.14	1.56±0.63	1.56±0.40	0.256
**Parameters associated with CVVHDF**						
Initiation time, d	1.77±2.32	1.79±2.40	1.80±2.33	1.72±2.31	1.70±1.72	0.649
Actual dose, mL/kg/hr	34.07±6.87	33.64±6.07	33.94±6.97	34.32±7.32	36.66±7.52	0.292
CRRTduration, d	5.45±5.93	5.30±5.25	5.52±6.39	5.51±6.04	5.30±4.59	0.891
Total ICU stay, d	15.12±40.29	12.34±15.63	18.34±59.36	13.86±20.03	9.60±8.87	0.432

Abbreviations: BMI, body mass index; CHF, congestive heart failure; CVA, cerebro vascular accident; MI, myocardial infarction; CKD, chronic kidney disease; LC, liver cirrhosis; MAP, mean arterial pressure; SOFA, sequential organ failure assessment; AKI, acute kidney injury; WBC, white blood cell; Hb, hemoglobin; Hct, hematochrit; PLT, platelet; TP, total protein; INR, international normalized ratio; CRRT, continuous renal replacement therapy; ICU, intensive care unit

### Short-Term Patient Mortality in Each Age Cohort

**Fig 1:**
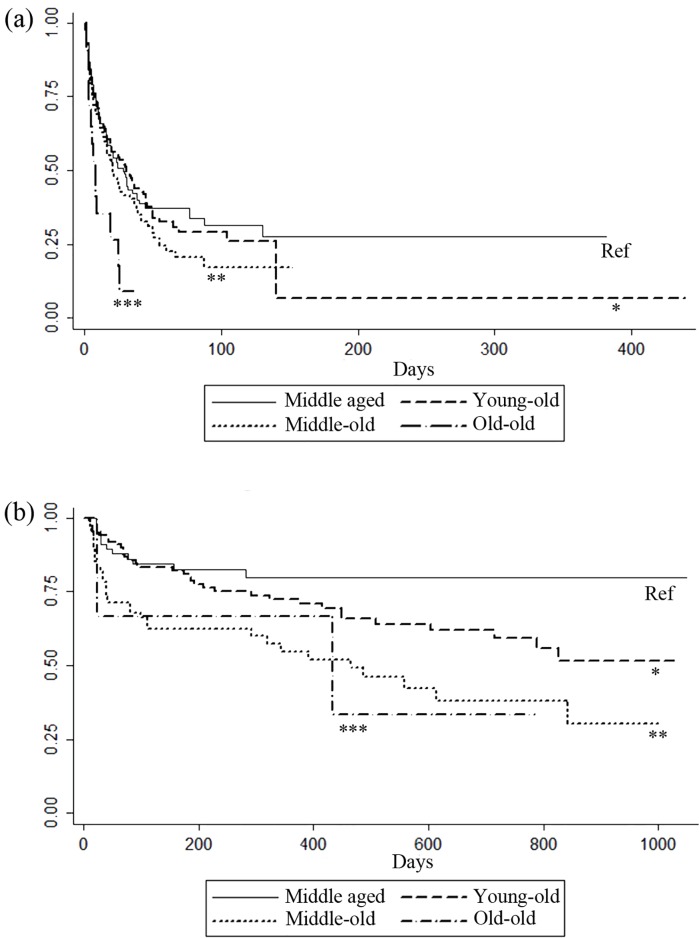
Short- (a) and long-term (b) Kaplan-Meier survival plot of patients with acute kidney injury who underwent continuous renal replacement therapy by different elderly groups. Survival difference was not observed in the young-old aged cohort when compared to the middle aged cohort in both short and long term follow up periods. (a) When compared to the middle aged cohort, survival difference was observed only in the old-old aged cohort. * Log rank between middle aged and young-old cohort, p = 0.699, ** Log rank between middle aged and middle-old cohort, p = 0.126, *** Log rank between middle aged and old-old cohort, p = 0.005 (b) When compared to the middle aged cohort, survival difference was observed in the middle–old and old-old cohort. * Log rank between middle aged and young-old cohort, p = 0.074, ** Log rank between middle aged and middle-old cohort, p = <0.001, *** Log rank between middle aged and old-old cohort, p = 0.049.

A total of 57.3% of the patients died during a mean hospital stay of 28.14±46.22 days. Death occurred in 55.0% of the middle-aged cohort, 54.6% of the young-old cohort, 61.7% of the middle-old cohort and 70.0% of the old-old cohort. When the Kaplan-Meier cumulative incidence plot was adjusted, the short-term mortality rate was significantly increased in the old-old aged cohort when compared to the middle aged cohort (Fig 1A). In the univariable and multivariable analyses, the short-term mortality rate significantly increased in the middle-old cohort (HR 1.485(1.090–2.021), p = 0.012) and the old-old cohort (HR 2.330(1.297–4.187), p = 0.005) compared with the middle-aged cohort. However, the mortality rate was not increased in the young-old cohort compared with the middle-aged cohort. The optimal cut off age to predict an increased short-term mortality rate in patients who underwent CRRT could not be calculated in this cohort (data not shown). In addition to age, a higher SOFA score, acidemia and prolonged prothrombin time at the time of CRRT initiation were independently associated with a higher short-term mortality rate in this cohort, Table 3.

**Table 3:** Factors associated with short term mortality.

	Univariated analysis			Mulivariated analysis		
Variables	HR	95% CI	P	HR	95% CI	P
Age	1.017	1.003–1.031	0.020			
Middle aged		Ref			Ref	
Young- old	1.044	0.790–1.379	0.763			
Middle- old	1.250	0.933–1.675	0.134	1.485	1.090–2.024	0.012
Old- old	2.488	1.406–4.404	0.002	2.330	1.297–4.187	0.005
Male	1.046	0.831–1.316	0.701			
BMI, kg/m^2^	0.994	0.966–1.022	0.675			
Diabetes	1.390	1.111–1.739	0.004			
Hypertension	1.566	1.239–1.979	<0.001			
CKD	1.676	1.300–2.162	<0.001			
CHF	1.377	1.096–1.731	0.006			
Old MI	0.927	0.715–1.202	0.570			
Atrial fibrillation	0.861	0.655–1.131	0.282			
Old CVA	0.948	0.735–1.224	0.684			
Liver cirrhosis,	1.292	0.892–1.872	0.175			
Cancer	1.626	1.277–2.071	<0.001			
Immunocompromised	2.005	1.427–2.818	<0.001			
SOFA score	1.123	1.091–1.157	<0.001	1.083	1.047–1.120	<0.001
Sepsis	1.060	0.851–1.320	0.604			
Use of vasopressin	1.940	1.524–2.472	<0.001			
Use of ventilator	1.503	1.192–1.895	0.001			
Acidemia(pH, mmHg)	0.089	0.036–0.220	<0.001	0.187	0.070–0.499	0.001
CRRT initiation time	1.033	0.996–1.072	0.085			
Delivered dose,	0.996	0.980–1.013	0.655			
Total protein, g/dL	0.804	0.714–0.906	<0.001			
Serum albumin, g/dL	0.749	0.619–0.908	0.003			
Serum BUN, mg/dL	0.995	0.991–0.999	0.012			
Serum Cr, mg/dL	0.892	0.839–0.948	<0.001			
Platelet,10E3/uL	0.998	0.996–0.999	0.001			
PT, INR	1.250	1.143–1.367	<0.001	1.276	1.150–1.417	<0.001

Foot note: Multivariable analysis was performed adjusted with age cohort, sex, presence of DM, HT, CKD,CHF, cancer, immunocompromised, SOFA score, pH, CRRT initiation time, serum total protein, serum albumin and prothrombin time. Abbreviations: BMI, body mass index; CKD, chronic kidney disease; CHF, congestive heart failure; MI, myocardial infarction; CVA, cerebro vascular accident; SOFA, sequential organ failure assessment; CRRT, continuous renal replacement therapy; BUN, blood urea nitrogen; Cr, creatinine; PT, prothrombin time

### Long-Term Patient Mortality in Each Age Cohort

Among the 240 survived patients, 238 were followed up for a mean duration of 362.74±314.98 days. The total follow up duration was not significantly different among the four age cohorts. During the follow-up period, death occurred in 17.6% of the middle-aged cohort, 33.3% of the young-old cohort, and 50.0% of both the middle- old and the old-old cohorts. The mortality rate significantly increased with increasing age (Fig 1B). In the univariable and multivariable analyses, long-term mortality rate significantly increased in the middle-old cohort (HR 3.757(1.838–7.679), p<0.001) and old-old cohort (HR 4.393(1.199–16.090), p = 0.025) compared with the middle-aged cohort. However, in the young-old cohort, the mortality rate did not significantly increase compared with the middle-age cohort. The optimal cut off age that predicted an increased long-term mortality rate in patients who underwent CRRT was 72 years old (S2 Fig). In addition to age, a low BMI, the presence of congestive heart failure, the presence of liver cirrhosis, prolonged prothrombin time and a history of sepsis at the time of CRRT initiation were independently associated with a higher long-term mortality rate, Table 4.

**Table 4:** Factors associated with longterm mortality.

	Univariated analysis			Mulivariated analysis		
Variables	HR	95% CI	P	HR	95% CI	P
Age						
Middle aged	Ref			Ref		
Young- old	1.828	0.946–3.530	0.073			
Middle- old	3.458	1.774–6.739	<0.001	3.757	1.838–7.679	<0.001
Old- old	3.583	1.009–12.726	0.048	4.393	1.199–16.090	0.025
Male	0.630	0.403–0.985	0.043			
BMI, kg/m^2^	0.910	0.848–0.977	0.009	0.912	0.846–0.984	0.018
Diabetes	1.305	0.837–2.034	0.240			
Hypertension	1.148	0.634–2.080	0.649			
CKD	1.475	0.951–2.287	0.082			
CHF	1.626	1.045–2.532	0.031	1.686	1.055–2.695	0.029
Old MI	0.855	0.519–1.409	0.539			
Atrial fibrillation	1.343	0.826–2.184	0.235			
Old CVA	0.950	0.576–1.565	0.840			
Liver cirrhosis	2.164	0.994–4.710	0.052	3.316	1.435–7.663	0.005
Cancer	1.246	0.658–2.358	0.500			
Immunocompromised	2.941	0.716–12.075	0.134			
SOFA score	1.031	0.959–1.109	0.401			
History of Sepsis	2.107	1.354–3.278	0.001	2.225	1.407–3.517	0.001
Use of vasopressin	1.289	0.829–2.005	0.259			
Use of ventilator	1.374	0.885–2.134	0.157			
Acidemia(pH, mmHg)	1.901	0.275–13.151	0.515			
Total protein, g/dL	0.965	0.750–1.241	0.778			
Serum albumin, g/dL	0.746	0.519–1.074	0.115			
Serum BUN, mg/dL	1.000	0.993–1.007	0.935			
Serum Cr, mg/dL	0.977	0.896–1.065	0.592			
PT, INR	1.235	1.021–1.493	0.029	1.349	1.103–1.648	0.004

Foot note: Multivariable analysis was performed adjusted with age, sex, BMI, CHF, liver cirrhosis, CKD and presence of sepsis and PT. Abbreviations: BMI, body mass index; CKD, chronic kidney disease; CHF, congestive heart failure; MI, myocardial infarction; CVA, cerebro vascular accident; SOFA, sequential organ failure assessment; BUN, blood urea nitrogen; Cr, creatinine; PT, prothrombin time

## Discussion

In this retrospective cohort study of elderly AKI patients who underwent CRRT, the short-and long-term mortality rate were increased in the middle-old and the old-old cohorts. However, in the young-old cohort, neither the short-term nor the long-term mortality rates were increased compared with the middle-aged cohort. Thus, although increasing age is an important risk factor for mortality in elderly AKI patients who underwent CRRT, the odds of dying do not increase in young-old populations aged between 65 and 74 years old compared with the middle-aged cohort.

Several previous nationwide surveys have reported that the incidence of dialysis-dependent AKI patients is increasing, and it is greater in the elderly population aged over 65 years old [8, 16]. Increased age is a well-known predictor of poor CRRT outcome [11, 17]. However, the definitions for elderly that were used in previous studies vary greatly[4], and there are no studies that reported short- and long-term CRRT outcome according to the age subgroup. As far as we know, this is the first study that described this issue. Furthermore, we calculated a cut off value for the prediction of increased long-term CRRT mortality in this cohort. The estimated cut off age was 72 years old, which is approximately 7 years older than the 65-year-old threshold commonly used to define the elderly population. Further validation tests in larger cohorts are needed for this cut off age.

In studies published from 1999 to 2005, elderly patients tended to be treated less intensively than their younger counterparts, and this fact was thought to be associated with the higher ICU mortality of elderly patients [18–20]. In our cohort that contained data collected from 2013 to 2015, the treatment intensities were not significantly different among the age cohorts. The percentages of ventilator and vasopressor users and the duration of ICU stay were not different. Additionally, in the operation of CRRT, the initiation time, actual delivered dose and CRRT operation duration were not different among the four different age cohorts. This trend was comparable to the recently published data from Peigne et al. who compared and found no relations between the treatment intensity and age in a medical ICU cohort [21]. A nationwide study from Denmark evaluated the time trend of incidence of AKI patients who required dialysis and found that the mean age of AKI patients who required dialysis was older and that the growth of the incidence rate was the greatest in elderly patients aged more than 75 years old [8]. Although this phenomenon might be mainly associated with demographic changes associated with rising longevity, it might reflect the changes in physicians’ attitudes regarding the more aggressive treatment of elderly AKI patients.

In addition to age, short-term mortality was largely associated with variables that indicated the severity of illness at baseline; i.e., a higher SOFA score, acidemia and a prolonged prothrombin time were significant factors that predicted increased short-term mortality, and many of these factors have been suggested as predictors of higher mortality in previous studies. Meanwhile, long-term mortality was associated with variables that indicated a patient’s basal health status, such as a lower BMI, the presence of congestive heart failure, the presence of liver cirrhosis and a history of sepsis.

In a normal population, obesity was shown to be associated with a poorer patient outcome of various conditions [22, 23]. However, in patients with chronic kidney disease, patients with end stage renal disease and elderly nursing home residents, obese patients showed better survival than normal and underweight patients [24–26]. This obesity paradox was also observed in AKI survivors who underwent renal replacement therapy. In a previous study that investigated patients with AKI who needed renal replacement therapy, post-ICU mortality was highest in patients with a BMI lower than 18.5 kg/m^2^ and lowest in patients with a BMI between 30 and 35 kg/m^2^[27]. A similar trend was found in our elderly AKI cohort. With each 1 kg/m^2^ decrease in BMI, the long-term mortality rate increased by 9% after adjustment for age, sex, the presence of congestive heart failure, liver cirrhosis, chronic kidney disease and a history of sepsis. However, we could not investigate the protective effect of being overweight in this population because our cohort was largely composed of underweight and under to normal weight patients. The mean BMI level of our cohort was 23.00±3.98 kg/m^2^, and there were only 20 (3.5%) patients with a BMI over 30 kg/m^2^, while patients with a BMI lower than 20 kg/m^2^ comprised 20.4% of the entire cohort. Thus, in our cohort, we identified that malnutrition was harmful to elderly AKI patients who underwent CRRT; however, we were unable prove the protective effect of obesity in elderly AKI patients who underwent CRRT.

Given the interplay of multiple factors of AKI patients who underwent CRRT with long-term mortality rate, we observed that a history of sepsis was important for the prediction of long-term CRRT mortality. To analyze the effect of sepsis on long-term patient mortality, we grouped AKI survivors into sepsis and non-sepsis groups according to the initial cause of CRRT. We found that patients who survived after sepsis had a 2.2-fold increased risk of mortality compared with elderly AKI patients who underwent CRRT without sepsis. This finding was consistent with previous studies that reported an increased risk of mortality after sepsis or ICU admission in an elderly or general population [28–31]. It is uncertain whether the increased risk of post-sepsis mortality in our patients was a simple reflection of the trajectory of pre-morbid condition or sepsis itself. Additionally, we could not evaluate the causal link between sepsis and mortality after hospital discharge due to the retrospective manner of this study. However, it is certain that elderly AKI patients who underwent CRRT due to sepsis need more careful management than patients without sepsis after the hospital discharge.

Our data had several limitations. First, we did not investigate the final renal outcome, and we could not evaluate the association between the progression of chronic kidney disease after AKI and long-term mortality. Second, the number of patients in the old-old cohort was relatively small compared with that in the other groups; thus, it was insufficient to obtain a complete conclusion for the old-old cohort. However, we did not exclude their data in the analysis because the short- and long-term CRRT outcome reports of the extremely old population were scarce, and thus, we thought it would provide meaningful information in the decision to apply CRRT in this extremely old population. Third, due to the retrospective manner of this research, selection bias and several un-charted comorbidities or events could play a role in the interpretation of short- and long-term mortality in this study. Despite these limitations, the wide array of parameters, which included detailed comorbidities, treatment histories and laboratory data, used in this study strengthens the results herein.

In summary, our study shows that compared with the middle-aged cohort, the middle-old and the old-old cohort had an increased short- and long term mortality rate. However, in the young-old cohort, neither the short-term nor the long-term mortality rate was increased. Thus, we should be similarly unhesitant to initiate CRRT in patients aged between 65 and 74 years as we are with their younger counterparts.

## Supporting Information

S1 Fig Flow diagram of the study. (TIF)

S2 Fig Superior long-term survival in patients aged more than 72 years old among acute kidney injury survivors who underwent continuous renal replacement. (a) The standardized log-rank statistics according to the age. The maximum of the standardized statistics is 4.168 at 72 years old, p = 0.001. (b) Kaplan-Meier survival plot of patients with acute kidney injury who underwent continuous renal replacement therapy for the long term patient survival showed superior patient survival in patients with 72 years old or less. Log rank, p<0.001.
(TIF)
