# Supplementary figures and images for: Short- and Long-Term Mortality Rates of Elderly Acute Kidney Injury Patients Who Underwent Continuous Renal Replacement Therapy
Source: PLoS One. 2016 Nov 22;11(11):e0167067. doi: 10.1371/journal.pone.0167067 (PMC5119822; doi:10.1371/journal.pone.0167067)

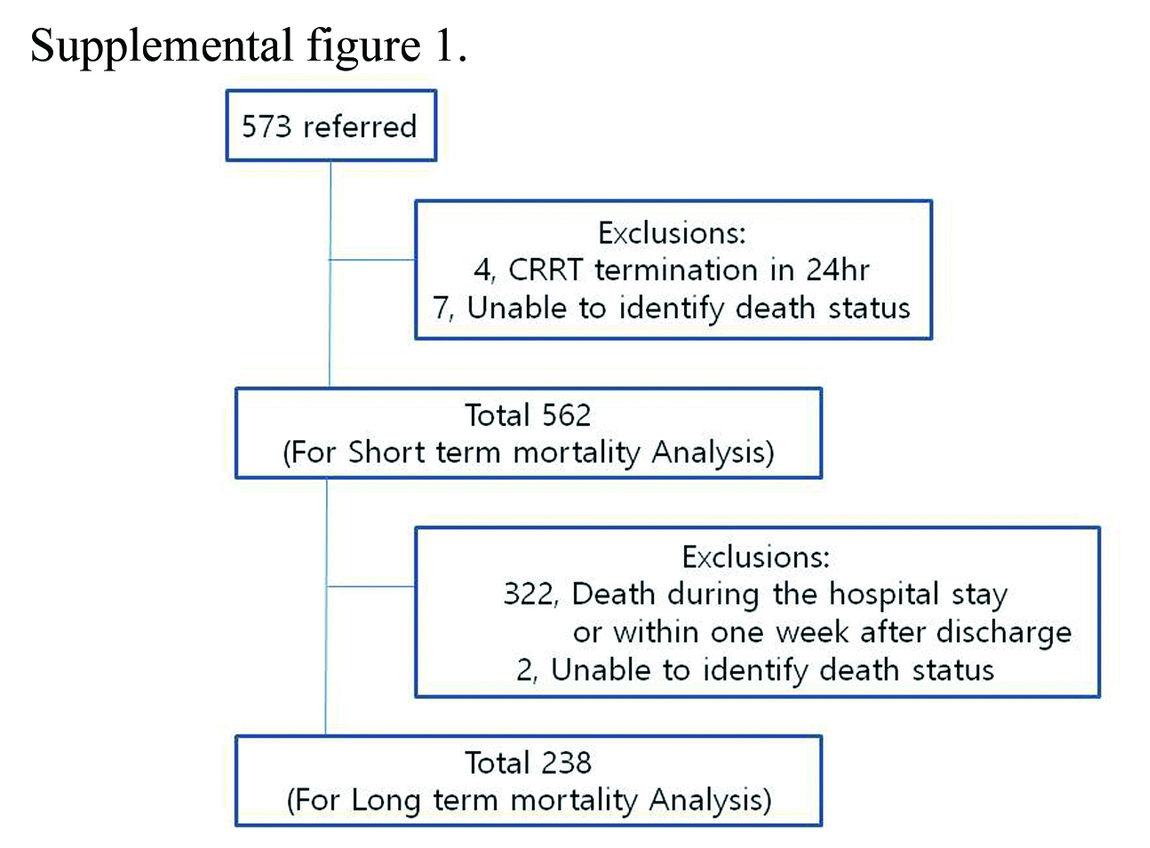

Supplement: S1 Fig — (TIF) [file pone.0167067.s001.tif]

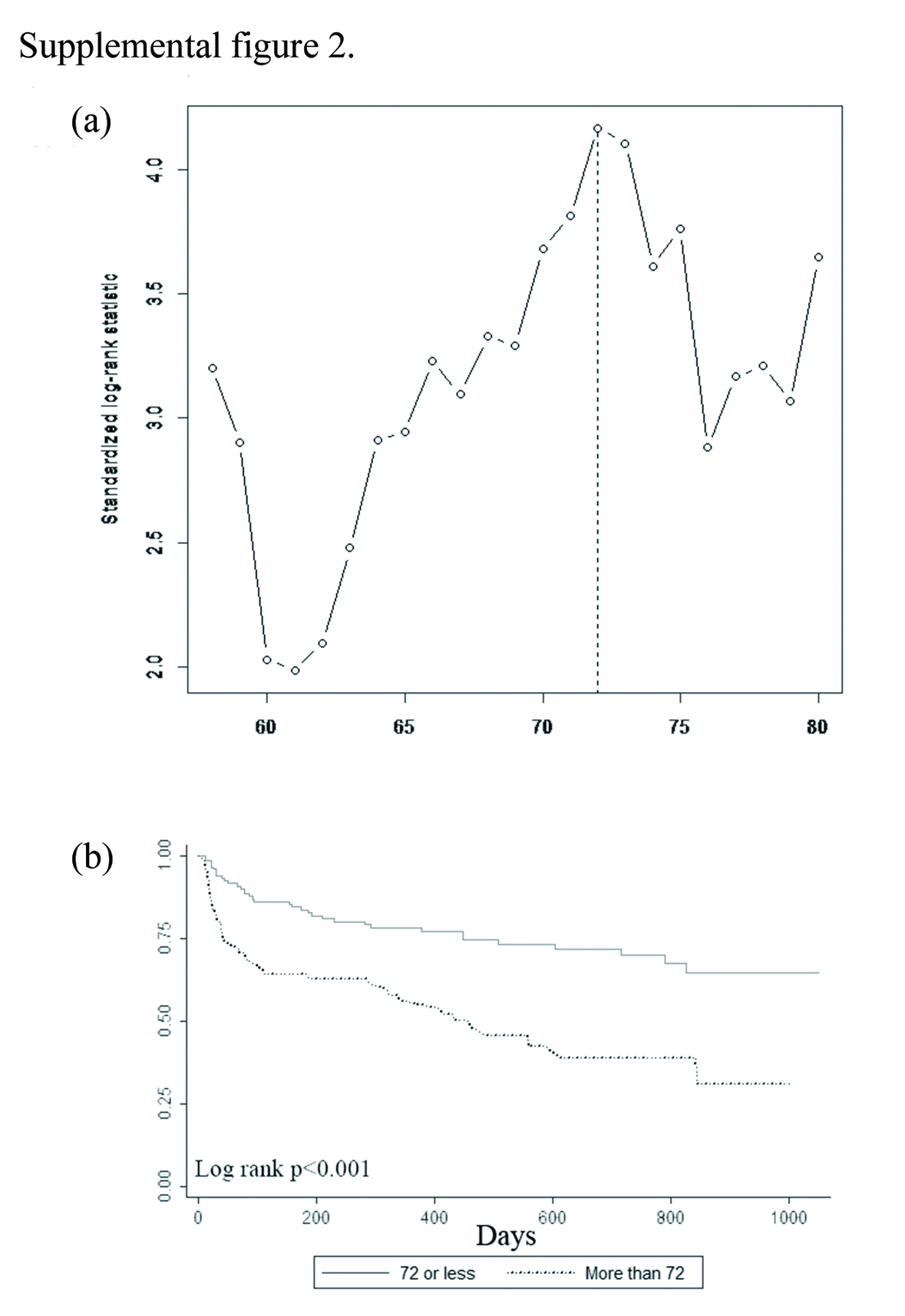

Supplement: S2 Fig — (a) The standardized log-rank statistics according to the age. The maximum of the standardized statistics is 4.168 at 72 years old, p = 0.001. (b) Kaplan-Meier survival plot of patients with acute kidney injury who underwent continuous renal replacement therapy for the long term patient survival showed superior patient survival in patients with 72 years old or less. Log rank, p<0.001. (TIF) [file pone.0167067.s002.tif]
